# Annotations

**Published:** 1900-03-03

**Authors:** 


### ANNOTATIONS.

### Sanatorium Treatment Gone Mad.

Divers medical journals have of late chosen to become almost hysterical in regard to the beauties and delights of a certain new sanatorium, the Villa Igiea by name, which has recently been established in the neighbourhood of Palermo. The founder of this establishment recently invited a number of medical celebrities from England to view its charms, and they have not failed on their return to speak well of the place where they received such hospitality; all of which is most proper, indeed only what was no doubt expected of them. Now, however, comes the funny part of the business. The medical director of this great sanatorium, this palatial mansion which will accommodate nearly 200 guests, is, it seems, also an inventor, who has added still one more to the long list of devices for curing consumption by inhalation! Thus, at a time in this world's history when we are patting each other on the back with no small effusiveness on having discovered that open air is the great cure for consumption, we are to be treated to the curious spectacle of people travelling all the way from England to Palermo, tempted there by the glorious air and the possibilities of outdoor life and all the rest of it, and then when they arrive finding themselves in an establishment where consumptives are being treated by being shut up for so many hours a day breathing the vapour of formic aldehyde! To all that is said about the charms of the climate of Palermo a willing assent may be given, and the Villa Igiea may be, as no doubt it is, one of the finest of European sanatoria; but fancy the bathos of the whole thing. The delicious climate, the equable temperature, the subtropical vegetation, the fountains, the marble terraces, the wealth of flowers, and the marvellous admixture of Italian art and modern sanitation—what could be more tempting and delightful, especially in view of modern theories which teach that the more of this beautiful air one gets the better? And then on arrival at this Elysium to shut oneself up in a room half suffocated with formic aldehyde of all things in this malodorous world!

### Baby Farming.

The urgent necessity of some better supervision being exercised over the very questionable business of the baby farmer is well shown by the revelations made in a recent case at the Central Criminal Court, in which a verdict of guilty was returned against a woman named Williams for the murder of a child which had been placed in her care. On the horrible details of this wretched case we do not now propose to make any comment. What is of interest is the disclosure made by the guilty woman as to the manner in which the business is conducted. According to her statement her method of proceeding was to advertise for children for adoption, and then to advertise again and get these unfortunate infants readopted by someone else for about half the sum that she had received. Thus we see displayed the villainy of the whole proceeding. We must not imagine that the mothers of the children whose fate it is to be farmed out are themselves murderers at heart. Not at all. They are anxious to be rid of an encumbrance and a shame; but maternal feeling is strong, and it is clear from the efforts made by the mothers in some of these cases to scrape together the money demanded for the "adoption" of their infants that they really do desire to find for them a "nice home." It is, in fact, upon the desire of the mothers to see their infants placed with decent and respectable people that the trade of the intermediary depends. How little do they think what is the fate of such children. The "nice home" is a mere receiving-house. The respectable woman in search of a child to comfort her solitude is a mere dealer in childen, who, by passing them on quickly so as to get rid of one before another comes in, is able to escape the somewhat feeble provisions of the Infant Life Protection Act, 1897; while the ultimate fate of the child is either slum life of the very lowest description, generally ending in death from "convulsions," or "marasmus," or murder of a more violent type, as in this case, in which, apparently as a cheaper proceeding than "re-adoption," the child was knocked on the head and dropped into the river. We fail entirely to appreciate the advantage of the various limitations which have been imposed on the applicability of the Infant Life Protection Act, limitations which have had the effect of rendering it practically impotent for good. The exemption of "institutions" established for the care of infants, and conducted "in good faith" and "for religious and charitable purposes," seems to us to serve no useful object, while the free scope given by the Act to the professional child murderer, so long as she only deals with one child at a time, seems very distinctly mischievous.

### The Reconstituted University of London.

According to the statutes made for the University of London by the Commissioners appointed under the University of London Act, 1898, all the medical schools in London, including that for women, in connection with hospitals, are in the list of the "first schools of the University," and in these schools it is provided that no student shall be admitted to examination for a medical or surgical degree who has not gone through the prescribed course of study in a medical school of the University, or a medical institution or school in the United Kingdom, or any dependency of the British Crown, or any foreign parts recognised by the Senate, with the approval of Her Majesty in Council. Power is also given to the Senate to grant an honorary or *ad eundem* degree in certain exceptional cases. It seems probable that at first the metropolitan medical student will not find that his lot is very much altered by the new arrangement. But the reconstituted University will be provided with large powers, which, if funds should be forthcoming, will enable it to exercise great influence over medical education in London, more especially in regard to the teaching of the earlier subjects. In fact, even from the beginning the University may make arrangements with the governing bodies of any schools of the University for the provision of common courses of instruction on such subjects, and for the interchange of students. But if funds should come in, and if wealthy donors should set the University on its feet with ample endowments, so that it could pay its professors and provide equipment for teaching purposes, then the influence of the University over the University schools would at once become paramount, and a complete revision and concentration of the earlier studies would become possible, much to the advantage of the student. As to the more special subjects it is left open to the Senate to make arrangements with the Royal Colleges to avoid duplicate examinations, so far as may be.

